# A systematic review of the effects of hepatitis B and C virus on the progression of liver fluke infection to liver cancer

**DOI:** 10.1186/s40794-023-00215-8

**Published:** 2024-03-15

**Authors:** Allison O’Rourke

**Affiliations:** George Mason University (Graduated), 4400 University Drive, Fairfax, VA 22030 USA

**Keywords:** Hepatitis, Liver flukes, Liver cancer, *Opisthorchis viverrini*, *Clonorchis sinensis*, Cholangiocarcinoma, Hepatocellular carcinoma

## Abstract

Hepatitis B and C virus, *Opisthorchis viverrini* and *Clonorchis sinensis,* are all individually known to put a person at increased risk for cholangiocarcinoma and hepatocellular carcinoma. This paper seeks to determine if there is any interaction between liver flukes and hepatitis virus infection that are known to put a person at an increased risk for cholangiocarcinoma and hepatocellular carcinoma collectively. This paper seeks to determine whether there is any publicly available articles in English that determine if having a hepatitis viral co-infection along with liver flukes would influence the risk of developing liver cancer. We followed PRISMA systematic review guidelines to conduct a literature review. Three manuscripts fit the search criteria. Two presented evidence in support of a synergistic relationship between liver fluke and viral hepatitis infection while the other found no relationship. One manuscript determined that the interaction between hepatitis B and *C. sinensis* did not have any significant risk of liver cancer. Studies found that HBV affected progression of co-infection to liver cancer but may have its own disease state worsened by presence of liver flukes. Only one paper was found that presented data on HCV, therefore no conclusion can be drawn due to the lack of evidence discovered. Of the studies, the conclusions and strength of the data were mixed. However, the stronger studies suggested a synergistic relationship between liver flukes and HBV to increase the risk of progressing to liver cancer.

**Supplementary Information:**

The online version contains supplementary material available at 10.1186/s40794-023-00215-8.

## Introduction

### Objectives

While the mechanism behind how liver flukes work to cause cholangiocarcinoma (CCA) and hepatocellular carcinoma (HCC) has been well studied, there is a dearth of peer-reviewed literature on how comorbidities such as viral hepatitis (B and C) affect the progression of CCA and HCC. The most vulnerable populations with comorbidities can benefit through more research on this topic. Once the disease progresses to CCA or HCC, it will almost always be fatal in these populations so prevention and treatment of liver flukes should be prioritized and identification of external factors that could be contributors.

In this paper, we examine the effect of Hepatitis B and C viruses on the progression of *O. viverrini* and *C. sinensis* to CCA or HCC. We conducted a systematic review to analyze the available literature and identified the gaps and limitations in the manuscripts which impede showing causality.

### Prevalence and geographic distribution

Liver fluke infections caused by *Opisthorchis viverrini*, *O. felineus*, and *Clonorchis sinensis* pose a major health risk to over 600 million people globally [7].

It is estimated that worldwide there are over 10 million people infected with *O. viverrini* and that 6–8 million of those individuals live in Thailand [24, 25]. *O. viverrini* is endemic to Southeast Asian countries, including Thailand, Lao People’s Democratic Republic, Vietnam, and Cambodia [8]. Even within Thailand the distribution of liver flukes is uneven with the north (19.3%) and northeast (15.7%) causing a high prevalence compared to the central (3.8%) and southern (0%) regions [6]. Khon Kaen province in northeast Thailand has the highest incidence liver fluke induced cholangiocarcinoma in the world [5, 16, 29].

It is estimated that globally over 35 million people are infected, and that 1.5–2 million people show symptoms or complications of *C. sinensis* [27]*. C. sinensis* infection is common in rural areas of Korea and China [8]. China has the largest population of infected people, which is estimated at 15 million [27].

Infection with food-borne parasites is common in these regions because uncooked cyprinoid fish are a staple of the diet. Liver fluke infection occurs when a human ingests raw freshwater fish that are fluke-infested [10]. Poor sanitation and sewage infrastructure in these countries also contribute to this problem. People infected with these liver flukes pass the eggs into the fresh water supply through the improper disposal of feces, which then leads to a continued cycle of infections [15].

In this article we are going to focus on *O. viverrini* and *C. sinensis* because they are predominantly found in Asia while O*. felineus* is mainly found in Italy, Germany, Belarus, Russia, Kazakhstan, and Ukraine [15]. These populations have different comorbidity burdens and access to healthcare which makes comparison insignificant.

### Mechanism for causing cancer

Most people who have *O. viverrini* and *C. sinensis* do not display any symptoms of infection. About 5%-10% of individuals, usually heavily infected, have symptoms of right upper quadrant abdominal pain, flatulence, and fatigue [12, 19, 28]. Long-term and heavy parasite count infection of liver flukes is associated with several different hepatobiliary diseases including cholangitis, obstructive jaundice, hepatomegaly, fibrosis of the periportal system, cholecystitis, and cholelithiasis [4, 23] .

Liver flukes are classified as a group 1 carcinogen as the mechanical, immunopathic, and secretory pathways of the parasite have been directly linked to causing cholangiocarcinoma (CCA) and Hepatocellular carcinoma (HCC) [11, 16, 25]. It is estimated that around one in six people who are infected with liver flukes will develop CCA or HCC and the prognosis is very poor with an almost 100 percent fatality rate [17].

Mechanically, the liver flukes use their suckers to bind to the walls of the biliary ducts which causes ulcers [18]. The liver fluke eggs can then become ensnared in those ulcers and can cause granulomatous inflammation of the periductal tissue. *C. sinensis*, which is larger in size than *O. viverrini*, can cause a partial bile duct obstruction resulting in bile stasis and an increase in biliary pressure. Additionally, the immune response to the parasite causes damage to the cells which contributes to the mutation of the DNA. Many different immune cells respond to the presence of the parasite and attack the region which it occupies. Finally, the parasite’s excretory/ secretory products promote immune mediated inflammation which also encourages DNA mutation. All this damage can cause inflammation and then eventually healing which repeats for the whole length of the infection. This cyclic process can eventually cause DNA mutations that lead to the development of CCA.

### Viral hepatitis and its role on the liver

Viral Hepatitis plays an important role in the global cancer scale as it is the second leading cause of cancer behind tobacco use [1, 2]. Hepatitis B virus (HBV) belongs in the *Hepadnaviridae* family and Hepatitis C virus (HCV)is a member of the *Flaviviridae* family. The viruses are estimated to chronically infect over 300 million people globally and about 2 billion people (1 in 3) have been infected at one point in their life (CDC, 2019). Most people who are infected with HBV/HCV can clear the virus but an estimated 5%-10% of adults and 90% of babies will develop chronic hepatitis [9]. Chronic hepatitis infection can cause liver damage that can lead to liver disease or liver cancer [9]. It is estimated that 60%-75% of global chronic hepatitis infections are found in Asia and are endemic in many parts of the country [13]. HBV/HCV has been circulating around Asian countries for many centuries and they historically have low rates of vaccination [20]. A large proportion of individuals with chronic HBV/HCV infection in these countries were infected at childbirth and it is common to see many individuals in one household with the virus [20].

Liver flukes are a neglected tropical disease and resources for treating it are limited in these Asian countries. Understanding the comorbidities that increase the rate of progression from liver fluke infection to CCA or HCC would allow for more targeted treatment of the highest risk populations.

## Methods

While performing this review we followed the PRISMA predefined systematic review guidelines. To summarize the following methods, we began by conducting a literature search based on the later established criteria. Then each article was screened to make sure that there were no duplicates and to make sure that they suited the inclusion criteria. Finally, the remaining articles were screened for their eligibility and included in the final analysis.

Inclusion criteria included the following parameters: (1) Any paper that looks at the following 3 diseases together (a)CCA or HCC (b) HBV or HCV (c) *O. viverrini* or *C. sinensis*; (2) Publications in English due to the language limitations of the reviewers; (3) published full text available.

Exclusion criteria included: (1) Non-human subjects; (2) Study with not reliably extracted, duplicate, or overlapping data; (3) Abstract-only papers as proceeding papers, conference, editorial, theses, and books; (4) Articles without available full text available, (5) Systematic review studies.

No exclusion was made on age, race, gender, and publication date. There were no exclusions included based on the study type, but it was required that it looked at the relationship between CCA or HCC, HBV or HCV, and *O. viverrini* or *C. sinensis*. There were articles that included all 3 of these words but did not examine them in relation to each other and those articles were excluded. The search included papers that were published from inception till 2021 which is when the review was conducted.

### Search strategy

**Fig. 1:**
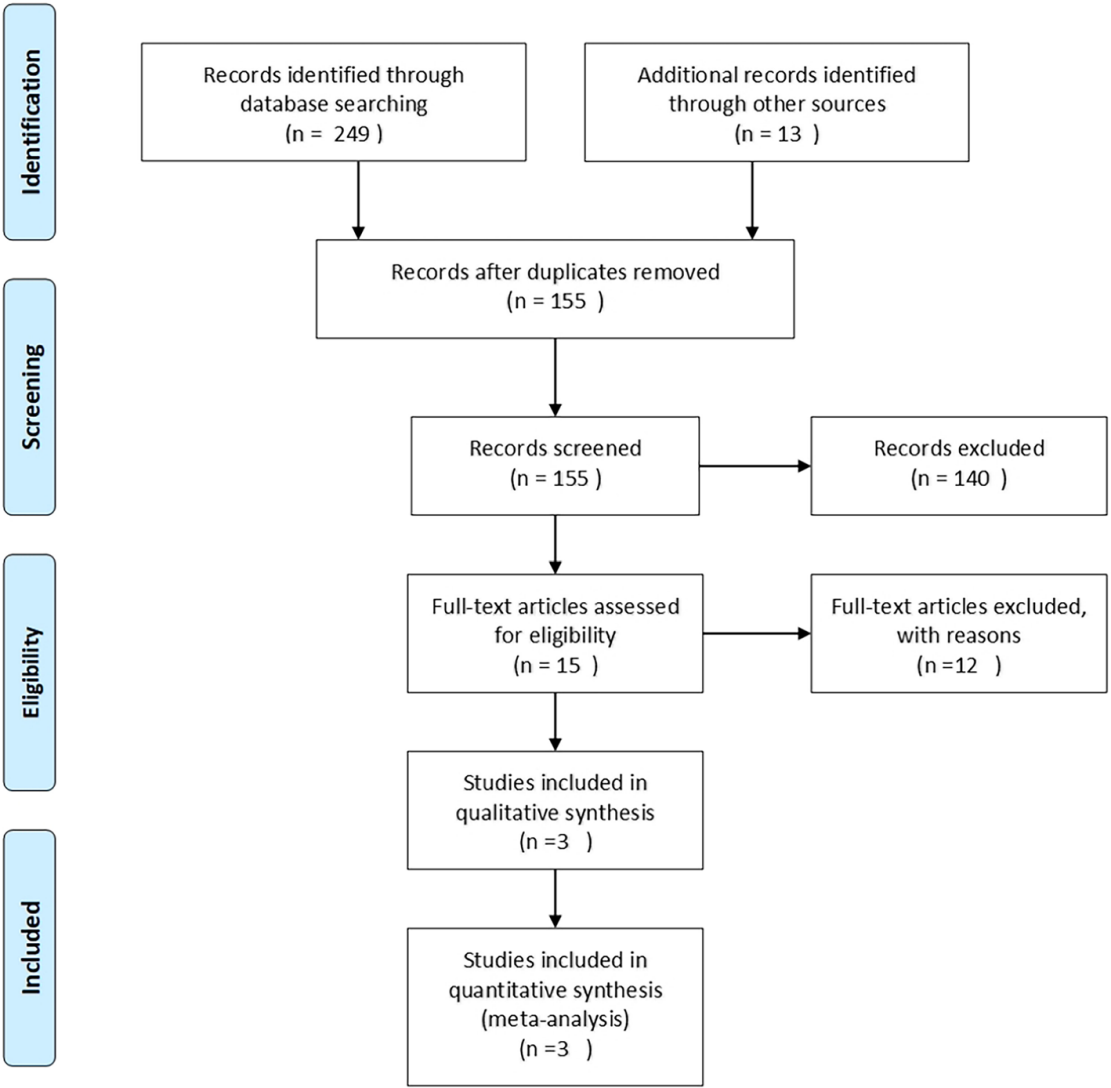
PRISMA flow diagram of search, screening, and identification of eligible manuscripts for the systematic review. This figure was adapted from [14]

A standard search strategy is used in PubMed, then later it is modified according to each specific database to get the best relevant results. Search strategies are constructed to include free-text terms such as the title or abstract. In PubMed the terms [tiab] and [MeSh] were used in the search to restrict the query to the title or abstract and to look for free text terms respectively. The following databases were searched: PubMed, Scopus, Cochrane, and Google Scholar. Figure 1 shows this process in the PRISMA flow diagram including the number of articles found at each stage.

The following search strings were used and adapted to each of the search databases:

- (“Opisthorchis viverrini” OR “O. viverrini” OR “Clonorchis sinensis” OR “C. sinensis” OR “Clonorchiasis” OR “Opisthorchiidae”)
- AND
- (“Hepatitis” OR “Hepatitis virus” OR “viral hepatitis” OR “Hep B” OR “Hep C” OR “HBV” OR “HCV”)
- (“liver cancer” OR “Hepatocellular carcinoma” OR “HCC” OR “Cholangiocarcinoma” OR “CCA” OR “gastrointestinal cancer” OR “GI Cancer” OR “primary liver cancer” OR “liver disease”)

Snowball sampling was also completed off the papers that were identified to fit the inclusion criteria. This included the papers that they cited as well as the papers that cited the identified articles.

There were no tools that were used to complete natural language processing or text frequency analysis. No automation tools were use in this literature review.

### Data screening

The primary author read the titles and abstracts of the full list of retrieved articles and kept those that either a) demonstrated relationships between liver flukes, Hepatitis virus, and liver cancer; or b) the purpose and results of the article could not be determined based on title or abstract alone.

A reviewer separately then analyzed the 15 full-text articles that had been found after duplicates had been removed, exclusion criteria had been applied, the articles were determined to be full length, and were published in English. The author and reviewer found three articles that fit the inclusion criteria and did not have any of the exclusion criteria. The other 12 articles and the reasons why they were excluded can be found in the Appendix. Data was then extracted by the author and checked by the reviewer to confirm that it was correct.

## Results

### Study selection

Our search strategy allowed us to identify 155 papers, of which 140 were excluded by title, abstract evaluation, and duplication. Duplicate entries were identified by considering the author, the year of publication, the title of the article, and the volume, issue, and page numbers of the source. In questionable cases, the abstract texts were compared. As a result, 15 studies were initially screened by reviewing the corresponding full-text papers. Then, 7 records were excluded due to lack of consistent evidence related to cancer, hepatitis, or liver flukes. Thus, 8 records were then assessed for eligibility according to the criteria outlined previously. Finally, 3 were eligible for our analysis. Type of source included 100% journal manuscripts.

### Analysis

As there were only three articles, the author extracted the data from each of the articles and conducted an analysis.

Information describing the studies analyzed are summarized in the Table 1, including author, publication year, study design, country, sample size, and evidence. We identified 1 case-series and 2 case-controls. The publication years ranged from 1996- 2017. Geographically 2 of the studies were conducted in China (66.7%) while 1 was conducted in Korea (33.3%). All the studies were conducted on humans, and they were all obtained after diagnosis with either viral hepatitis, liver cancer, or liver fluke infection.

**Table 1:** Manuscripts included in systematic review

Authors	Publication Year	Study Design	Sample Size	Country	Hepatitis Type	Liver Fluke	Cancer	Reference
Li, et al.	2016	Case- control	701	China	HBV and HCV	*C. sinensis*	HCC and CCA	(Li, Dong, Huang, Chen, Kong, Sun, Yu and Xu, 2016) [8]
Shin, et al.	1996	Case–control	650	Korea	HBV	*C. sinensis*	HCC and CCA	(Shin, et al., 1996) [22]
Shi, et al.	2017	Case-series	20	China	HBV	*C. sinensis*	HCC and CCA	(Shi, et al., 2017) [21]

The articles that were written by Shi et al., and Li et al., found that there was some correlation between viral Hepatitis and an increased risk for the progression of liver fluke infection to liver cancer [21]. They both found that HBV infection can not only affect that progression but also may have its own disease state worsened by the presence of liver flukes. Each of the papers concluded that the presence of liver fluke alongside viral hepatitis was accompanied by an increased HBV viral load, which in turn itself is a large risk for the development of HCC and CCA. Shi and colleagues’ study found that *C. sinensis* infection was a strong risk factor for CCA and HCC as 18 of the 20 patients identified had flukes in their stool [21]. Shi and colleagues’ paper also found that co- infection is commonly found in liver cancer patients with 7 out of the 20 (35%) of the CCA and HCC patients co-infected with liver flukes and HBV [21]. Li and colleagues’ paper noted that there were weaker liver function indicators and higher HBV DNA titers in patients who were dual-infected rather than mono-infected with HBV or *C. sinensis.* Liver function was measured using Alanine transaminase (ALT), Aspartate transaminase (AST), and total bilirubin (TB) numbers. Li and colleagues’ paper also supported the conclusion of a synergistic effect by showing that a combination anti-viral and anti-parasitic treatment had a much higher success rate when compared to an anti-viral treatment of HBV in patients who were dual-infected. Shi et al., and Li et al., both concluded that *C. sinensis* metabolites may directly enhance HBV replication and increase liver transaminases levels which can be interpreted as the co-infection of *C. sinensis* and HBV causing an aggravated disease state. All of the data in these two papers were able to support a compounding effect of HBV infection and *C. sinensis* causing an increased risk for HCC and CCA.

The final paper, written by Shin, was a case–control that looked at the combination of many different factors and their effect on liver cancer [22]. This paper looked at the combination of viral hepatitis and *C. sinensis* on an individual’s risk for CCA and HCC. The authors found that the presence of having a hepatitis B surface antigen (HBsAg) positive test led to a statistically significant relative risk (RR) of 87.4 for the development of HCC. They also were able to show that anti-HCV positivity, *C. sinensis* in the stool, transfusion history, and liver fluke history all had relative risks over 2 for both HCC and CCA. The RR for *C. sinensis* in the stool, hepatitis history, and liver fluke history were statistically significant for development of CCA. When the model included interaction terms for hepatitis virus and *C. sinensis* in the stool and were compared with the full model by the log likelihood ratio test they found that it did not have any significant risk of liver cancer. They ended up concluding that HBV and HCV were independent risk factors of the development of HCC. While liver fluke infection was a risk factor for CCA, there was not significant information to support any synergistic effect between liver fluke infection, hepatitis virus, and liver cancer.

There can be no clear conclusions drawn about HCV as there was only one paper that examined it. With only one study available, not enough data is present to provide analysis on the effect that HCV has on liver cancer progression in liver fluke patients.

### Analysis of bias

To minimize the bias that can be present from the author, an independent review was conducted by a second reviewer and then a consensus was reached. Only studies that were found to have a valid level of association were included in the study. Bias within each paper was analyzed by the author and a reviewer. Selection bias, information bias, recall bias, and assessment of certainty in body of evidence were added into Table 2.

**Table 2:** Analysis of bias for each paper selected for review

Paper	Selection bias	Information bias	Recall bias	Assessment of certainty in body of evidence
Li et al. 2016 [8]	Not likely. All patients were selected based upon objective measurements of Hepatitis B infection or Liver fluke infection	Not likely, objective measurements of Hepatitis B infection or Liver fluke infection are standard and not subjective (i.e., there are clear markers of infection)	N/A	Generalizability could be an issue because patients with these factors were excluded from analysis: “those co-infected with HIV, hepatitis A, C, D and E, those with type I and type II diabetes, those co-infected with *Schistosoma japonicum*, or *Schistosoma mansoni* or other parasites, and those with alcoholic liver, autoimmune diseases, cholestasis, serious heart diseases and pregnant women.”
Shin et al. 1996 [22]	Not likely. All patients were selected based upon objective measurements of Hepatitis B infection. Assessment of liver fluke infection was not described, but selection based upon observation in stool samples is unlikely related to known status of Hepatitis infection	Liver fluke infection could have been misclassified. Details were not described in Methods section	N/A	Relative risk estimates are very high, but the confidence intervals are wide. A larger sample size could have strengthened the certainty of relationships
Shi et al. 2017 [21]	Not likely. All patients were selected based upon objective measurements of Hepatitis B infection or Liver fluke infection	Although the study used objective measurements of Hepatitis B infection or Liver fluke infection, there was not a true control group. Of the 20 carcinoma patients, only one patient was not co-infected with liver fluke	N/A	Small sample size and lack of a control group limits certainty of evidence

## Discussion

Overall, there is not enough sufficient evidence to draw strong conclusions. There are strengths and weaknesses of each paper that can be used during the analysis.

Shi’s paper was a case series which observed 20 clinical liver cancer cases. There is no control group in which the data can be compared. The size of the case-series is also only 20 which is not a very large sample size and can cause bias in the data. The cases were only taken from individuals who visited the Hengxian People’s Hospital which could potentially cause some level of selection bias. The researchers also were not able to exclude factors that impaired the liver in other ways such as alcohol consumption, hepatitis A infection, etc. which could have impacted the liver in other ways. The paper states that they were very limited in their funding and manpower which led to such a narrow scope of investigation and a small number of clinical cases. Despite these limitations though, they were still able to conclude that co-infection with liver flukes and HBV led to an increase in risk for HCC and CCA.

The papers by Shin and colleagues and Li and colleagues, respectively, had much larger sample sizes and a more diverse population [22, 8] . Li and colleagues' paper had 701 participants and Shin and colleagues’ paper had 650 participants. Both papers were case–control studies which are considered more reliable because they follow the experimental design and have a control group which allows for better comparison. One of the limitations in Shin and colleagues’ paper though was the smaller CCA population size. They only had 41 CCA cases to study compared to the 203 HCC cases. Shin and colleagues’ research is outdated though with data being collected between 1990 and 1993. There has been a lot of development in technology and treatments since that time so the data may not be as reliable as data that was more recently collected and analyzed. Since this paper was published there have been other publications that dispute some of the findings found. In the paper they concluded that HBsAg positivity does not increase your risk for CCA, but there has now been recent evidence that shows HBsAg is the leading cause of liver cancer worldwide [26]. There has also been evidence found that the co-infection of HBV and HCV do have a synergistic effect on an individual’s risk for the development of HCC [3]. Since the findings in Shin and colleagues’ paper have since been disputed, their discussions and conclusions should be interpreted cautiously.

The two papers by Shi and colleagues and Li and colleagues concluded that that there was sufficient evidence to determine a compounding relationship between liver fluke and HBV infection. All of the papers are able to show that HBV and *C. sinensis* in the stool individually increase your risk for CCA and HCC, but Shi et al., and Li et al., concluded they influence each other. Li et al., found that liver function, which was measured by ALT, AST, and TB levels, was significantly higher in the liver fluke and HBV co-infected group when compared to the HBV mono-infected group. Li et al., also found HBV DNA log copies were also significantly higher in the co-infected group. As increased HBV viral load was shown in all 3 papers as a strong predictor for HCC and CCA it can be assumed that there is a compounding effect as *C. sinensis’* metabolites increases HBV proliferation. Most of the data from this literature review supports the hypothesis that there is a compounding or synergistic effect of co-infection of *C. sinensis* and HBV infection and an individual’s risk of HCC or CCA development.

There were some large gaps in the research that were found when conducting this review. The first that was identified was that there were no papers that looked at *O. viverrini* in detail. All the papers used evidence that only pertained to *C. sinensis* and mentioned that *O. viverrini* was also potentially a factor. There was only one paper that analyzed HCV which led to no conclusions being drawn. With such limited data, HCV may be prioritized for further investigation. Research needs to be done to better identify how both liver flukes are affected by HBV/HCV. It can also be seen that there is a lack of research in this field in general. There were only 3 papers that could be identified that looked at this issue. There is a lack of understanding of how other factors affect this progression. It is important that more research be done to identify the effect that viral hepatitis, as well as other comorbidities, have on the progression of liver fluke infection to liver cancers.

## Conclusion

This analysis seeks to pull together all the research that looks at the effect of HBV/HCV on the progression of *Opisthorchis viverrine* and *Clonorchis sinensis* to HCC and CCA. It also seeks to highlight pertinent gaps in the research and to identify opportunities to develop programs that could help address these needs. Of the few studies, the conclusions and strength of the data were mixed. However, the stronger studies suggest that there is a synergistic relationship between liver flukes and HBV/HCV to increase the risk of progressing to liver cancer. Further research into the interactions between the two diseases may provide an insight into the biochemical and mechanical mechanisms that make the liver vulnerable to cancer. In the regions where liver flukes are found there are a number of other diseases that affect the liver such as diabetes, cirrhosis, and non-alcoholic fatty liver disease that have a high prevalence. Research into the relationship between these comorbidities and liver flukes may provide further insight into the mechanisms of disease progression.

### Supplementary Information

**Additional file 1: Table 2. **List of the full text articles that were excluded after assessment for eligibility. Inclusion and exclusion criteria were assessed based on the title and abstract of each article and the following were deemed not applicable to this review.

## Data Availability

• The three main articles analyzed in this paper are available at the following websites. • https://journals.plos.org/plosntds/article?id/10.1371/journal.pntd.0004806 • https://link.springer.com/article/10.1007/s00436-017–5572-1 • https://academic.oup.com/ije/article/25/5/933/689218
