# Supplementary material for: A systematic review of the effects of hepatitis B and C virus on the progression of liver fluke infection to liver cancer
Source: Trop Dis Travel Med Vaccines. 2024 Mar 15;10:6. doi: 10.1186/s40794-023-00215-8 (PMC10941421; doi:10.1186/s40794-023-00215-8)
Supplement: Supplementary file 1 — Additional file 1: Table 2. List of the full text articles that were excluded after assessment for eligibility. Inclusion and exclusion criteria were assessed based on the title and abstract of each article and the following were deemed not applicable to this review. [file 40794_2023_215_MOESM1_ESM.docx]

**Appendix**

Table 2. List of the full text articles that were excluded after assessment for eligibility. Inclusion and exclusion criteria were assessed based on the title and abstract of each article and the following were deemed not applicable to this review.

| **Title** | **Authors** | **Explanation of Exclusion** |
| --- | --- | --- |
| Epidemiology of cholangiocarcinoma: An update focusing on risk factors | Hai-Rim Shin Jin-Kyoung Oh Eric Masuyer Maria-Paula Curado Veronique Bouvard Yue-Yi Fang Surapon Wiangnon Banchob Sripa Sung-Tae Hong | The article did not show a relationship between the three diseases. Article is already a systematic review. |
| Liver cancer: Descriptive epidemiology and risk factors other than HBV and HCV infection | Shu-ChunChuanga, Carlo LaVecchia, PaoloBoffettaa | Article did not show a relationship between the three diseases |
| High prevalence of Clonorchis sinensis infections and coinfection with hepatitis virus in riverside villages in northeast China | Yanhang Gao, Yanqing Li, Xiaowen Liu, Tong Zhang, Ge Yu, Yang Wang, Ying Shi, Xiumei Chi, Xiaomei Wang, Xiuzhu Gao, Ruihong Wu, Yingyu Zhang, Lei Hang, Shijuan Sun, Yazhe Guan, Ying Xu, Jing Meng, Xu Liu, Chang Jiang, Heming Ma, Liting Luo, Qi Yan, Xin Yin, Fei Peng, Yixiao Zhi, Weige Qu, Xia Zhang, Tianqi Ren, Lili Liu, Jinming Zhao, Feiyu Zhang, Adila Yakepu, Yu Pan, Hongqin Xu & Junqi Niu | Article did not show a relationship between the three diseases |
| The role of infection by opisthorchis viverrini, hepatitis B virus, and aflatoxin exposure in the etiology of liver cancer in thailand. A correlation study | Petcharin Srivatanakul PhD ,D. Maxwell Parkin MD ,Yuan‐Zhou Jiang PhD ,Myriam Khlat PhD ,U‐Thai Kao‐Ian MD ,Sineenat Sontipong BS, Christopher Wild PhD | Article did not show a relationship between the three diseases |
| Current status of infection-related gastrointestinal and hepatobiliary diseases in Thailand | P Kullavanijaya , P Tangkijvanich, Y Poovorawan | The article did not show a relationship between the three diseases. Article is already a systematic review. |
| Evaluation of Risk Factors and Clinicopathologic Features for Intrahepatic Cholangiocarcinoma in Southern China: A Possible Role of Hepatitis B Virus | Ning-fu Peng MMed, Le-qun Li PhD, Xiao Qin MMed, Ya Guo PhD, Tao Peng PhD, Kai-yin Xiao PhD, Xi-gang Chen MMed, Yu-feng Yang MMed, Zhi-xiong Su PhD, Bin Chen PhD, Ming Su PhD & Lu-nan Qi MMed | Article did not show a relationship between the three diseases |
| Epidemiology of liver cancer: an overview | Petcharin Srivatanakul 1, Hutcha Sriplung, Somyos Deerasamee | The article did not show a relationship between the three diseases. Article is already a systematic review. |
| Cholangiocarcinoma and Clonorchis sinensis infection: a case-control study in Korea | Dongil Choi 1, Jae Hoon Lim, Kyu Taek Lee, Jong Kyun Lee, Seong Ho Choi, Jin Seok Heo, Kee-Taek Jang, Nam Yong Lee, Seonwoo Kim, Sung-Tae Hong | Article did not include all three diseases in their analysis and does not show a relationship between the three diseases |
| Liver Fluke-Associated Biliary Tract Cancer | Piyapan Prueksapanich, Panida Piyachaturawat, Prapimphan Aumpansub, Wiriyaporn Ridtitid, Roongruedee Chaiteerakij, and Rungsun Rerknimitr | Article did not include all three diseases in their analysis and does not show a relationship between the three diseases |
| Fraction and incidence of liver cancer attributable to hepatitis B and C viruses worldwide | Delphine Maucort‐Boulch, Catherine de Martel, Silvia Franceschi, Martyn Plummer | Article did not include all three diseases in their analysis and does not show a relationship between the three diseases |
| Liver Fluke Induces Cholangiocarcinoma | Banchob Sripa ,Sasithorn Kaewkes,Paiboon Sithithaworn,Eimorn Mairiang,Thewarach Laha,Michael Smout,Chawalit Pairojkul,Vajaraphongsa Bhudhisawasdi,Smarn Tesana,Bandit Thinkamrop,Jeffrey M Bethony,Alex Loukas,Paul J Brindley | Article did not include all three diseases in their analysis and does not show a relationship between the three diseases |
| Roles of liver fluke infection as risk factor for cholangiocarcinoma | Paiboon Sithithaworn, Puangrat Yongvanit, Kunyarat Duenngai, Nadda Kiatsopit, Chawalit Pairojkul | Article did not include all three diseases in their analysis and does not show a relationship between the three diseases |
